# Mapping Integron-Associated AMR Genes in Whole Genome Sequences of *Salmonella* Typhimurium from Dairy Cattle

**DOI:** 10.3390/antibiotics14070633

**Published:** 2025-06-21

**Authors:** Sami Ullah Khan Bahadur, Nora Jean Nealon, Joshua B. Daniels, Muhammad Usman Zaheer, Mo Salman, Sangeeta Rao

**Affiliations:** 1 Animal Population Health Institute, College of Veterinary Medicine and Biomedical Sciences, Colorado State University, Campus Stop 1644, Fort Collins, CO 80523, USA; sami.bahadur@colostate.edu (S.U.K.B.); zaheer.muhammad.usman@gmail.com (M.U.Z.); mo.salman@colostate.edu (M.S.); 2 Department of Diagnostic Medicine and Pathobiology, Shreiber School of Veterinary Medicine, Rowan University, Glassboro, NJ 08028, USA; njnealon@gmail.com; 3 Department of Microbiology, Immunology and Pathology, College of Veterinary Medicine and Biomedical Sciences, Colorado State University, Fort Collins, CO 80523, USA; josh.daniels@colostate.edu; 4 Food and Agriculture Organization of the United Nations, Regional Office for Asia and the Pacific, Bangkok 10200, Thailand

**Keywords:** *Salmonella* Typhimurium, antimicrobial resistance genes, class-1 integrons, cattle, plasmids, genomic location, whole genome sequencing

## Abstract

**Background:** Antimicrobial resistance (AMR) is a critical global health threat, with AMR *Salmonella enterica* serovar Typhimurium strains being a major foodborne pathogen. Integrons, a type of mobile genetic element, capture and transfer resistance genes, thereby playing a role in the spread of AMR. **Objectives:** This study aimed to characterize the locations of integrons carrying AMR genes within the whole genomes of 32 *Salmonella* Typhimurium isolates collected from dairy cattle by two U.S. Veterinary Diagnostic Laboratories between 2009 and 2012. **Methods:** Class I integrons were sequenced from PCR-amplified products. DNA was extracted, quantified, barcoded, and sequenced on the Illumina MiSeq platform. Whole genome sequences were trimmed and assembled using the SPAdes assembler in Geneious Prime^®^, and plasmids were identified with the PlasmidFinder pipeline in Linux. Integron locations were determined by aligning their sequences with whole genome contigs and plasmids, while AMR genes were identified through BLAST with the MEGARes 3.0 database and confirmed by alignment with isolate, plasmid, and integron sequences. Statistical analysis was applied to compare the proportions of isolates harboring integrons on their chromosome versus plasmids and also to examine the associations between integron presence and AMR gene presence. **Results:** Seven plasmid types were identified from all isolates: IncFII(S) (n = 14), IncFIB(S) (n = 13), IncC (n = 7), Inc1-I(Alpha) (n = 3), and ColpVC, Col(pAHAD28), and Col8282 (1 isolate each). Of the 32 isolates, 16 (50%) carried at least one size of integron. Twelve of them carried both 1000 and 1200 bp; 3 carried only 1000 bp and 1 carried 1800 bp integrons. Of the 15 isolates that carried 1000 bp integron, 12 harbored it on IncFIB(S) plasmids, 2 on IncC plasmids, and 1 on the chromosome. The 1200 bp integrons from all 12 isolates were located on chromosomes. There were significant positive associations between the presence of integrons and the presence of several AMR genes including *sul1*, *aadA2*, *blaCARB-2*, *qacEdelta1*, *tet(G),* and *floR* (*p* < 0.05). AMR genes were located as follows: *aadA2* on IncFIB(S) and IncC plasmids; *bla_CMY-2_* on IncC plasmid; *qacEdelta1* on IncFIB(S), IncC, and chromosome; *bla_CARB-2_*, *floR*, *tet(A)* and *tet(G)* on the chromosome. **Conclusions:** The findings highlight the genomic and plasmid complexity of *Salmonella* Typhimurium which is impacted by the presence and location of integrons, and this study provides genomic insights that can inform efforts to enhance food safety and protect both animal and public health.

## 1. Introduction

Antimicrobial resistance (AMR), the ability of microorganisms to persist or grow in the presence of drugs designed to inhibit or kill them, was identified by the WHO in 2019 as a top global health threat and it has contributed to an estimated 4.95 million deaths [1,2]. Although AMR emerged shortly after the introduction of the first effective antimicrobial agent [3], it is a multifaceted and dynamic issue driven by various factors, with antimicrobial use in human, agriculture, and animal production sectors serving as a major contributor to its rapid increase and global dissemination [4]. Among the pathogens contributing to this crisis, non-typhoidal salmonellosis, driven largely by *Salmonella enterica* serovar Typhimurium (*S.* Typhimurium), is particularly significant [5]. *S*. Typhimurium, due in part to its zoonotic capabilities, is a leading cause of foodborne illnesses and diarrheal diseases worldwide in people and animals [6]. Non-typhoidal *Salmonella* is responsible for approximately 93.8 million cases of human gastroenteritis and 155,000 deaths globally each year, with *Salmonella enterica* itself accounting for 57,000 deaths out of the 1.53 million reported cases [7]. In the United States alone, *Salmonella* infections amount to approximately 1.35 million cases, 26,500 hospitalizations, and 420 deaths annually [8]. The zoonotic nature and prevalence of *S.* Typhimurium in people, retail meats, and animals [9] make it a valuable model organism for studying the persistence and transmission of AMR bacteria through the food chain.

Due to public health importance and widespread AMR, the monitoring of *S.* Typhimurium is essential. In the United States, *S.* Typhimurium outbreaks and genomic epidemiology are tracked through collaborations with the National Antimicrobial Resistance Monitoring System, managed through the US Food and Drug Administration [10]. The public health risk of AMR *S.* Typhimurium is further increased due to its ability to spread through multiple routes, including via foodborne and environmental transmission [11]. Dairy cattle can serve as reservoirs and shed *S.* Typhimurium into the environment even when they appear healthy [12]. Containment of antimicrobial-resistant *S.* Typhimurium in dairy cattle may subsequently decrease the consequences of the disease in humans [13]. However, the acquisition of antimicrobial resistance genes (ARGs) accelerates the spread of AMR between and among bacterial and host species. Among the mechanisms driving AMR, horizontal gene transfer (HGT) plays a more critical role than genetic mutations in the spread and persistence of resistance [14]. HGT facilitates the exchange of ARGs between bacteria of the same generation through mobile genetic elements such as plasmids, transposons, and integrons, and later vertical transmission ensures their inheritance to subsequent generations through cell division [14].

Integrons are mobile genetic elements that capture gene cassettes and they often carry AMR genes [15]. Integrons encode for an integrase enzyme (IntI) that catalyzes site-specific nucleotide recombination at attC regions and facilitates the integration and expression of these gene cassettes [15]. By enabling the acquisition, expression, and spread of ARGs, integrons contribute significantly to the evolution of AMR. The mobility and stability of integrons, influenced by their genomic location (e.g., chromosomal versus plasmid), further determine their role in facilitating horizontal gene transfer and sustaining AMR within bacterial populations [16]. In addition, integrons have also been recognized as capable of capturing and mobilizing virulence factors, thereby enhancing the pathogenic potential of bacterial hosts [17]. When AMR genes and virulence factors are dually expressed by a pathogenic microorganism, this can result in a drug-resistant infection that is simultaneously difficult to treat with conventional antimicrobial agents.

It has been established that the presence of integrons in *S.* Typhimurium isolates from humans, cattle, poultry and swine has a significant association with phenotypic multidrug resistance [18]. Furthermore, an examination of *S.* Typhimurium isolated from poultry identified differences in AMR gene carriage across isolates with versus without integrons and showed that integrons can co-localize on plasmids with AMR genes to create unique resistance gene profiles [19]. Although *S.* Typhimurium isolated from cattle has distinct AMR patterns when compared to those of people and poultry [20,21], few studies have specifically investigated integron-mediated resistance in bovine isolates. In particular, there is a limited understanding of whether integrons are located on the chromosome or plasmids, and how this influences the co-occurrence and potential transfer of AMR genes. This study addresses this gap by analyzing the genomic location of integrons and associated AMR genes in *S.* Typhimurium isolates from dairy cattle.

The primary objective of this study was to investigate how the presence and genomic location (chromosome versus plasmid) of integrons in *S.* Typhimurium isolates from cattle influence the distribution of AMR genes within and between isolates. Determining the location of integrons on chromosomes versus plasmids within *S.* Typhimurium genome will be crucial in understanding the spread, persistence and evolution of AMR genes and virulence factors. Given the role of integrons in the acquisition and dissemination of AMR, AMR genes are likely harbored near integrons within the genome of *S.* Typhimurium. Moreover, the chromosomal and plasmidic locations of integrons and AMR genes influence their expression and potential for horizontal transfer between bacteria [14]. By comparing AMR gene profiles of *S*. Typhimurium that carry integrons on plasmids versus the chromosome, we seek to clarify how these genetic differences influence the dissemination and persistence of AMR.

## 2. Results

### 2.1. Serotype Confirmation

Following both traditional slide agglutination, 33 isolates were confirmed as S. Typhimurium. However, following concurrent Seqsero2 analysis, 32 isolates were confirmed as *S.* Typhimurium and one isolate was identified as *Salmonella* Agona. The 32 isolates that were confirmed as *S.* Typhimurium by both methods were included in the downstream analysis.

### 2.2. Integrons and Plasmids Identified

**Figure 1:**
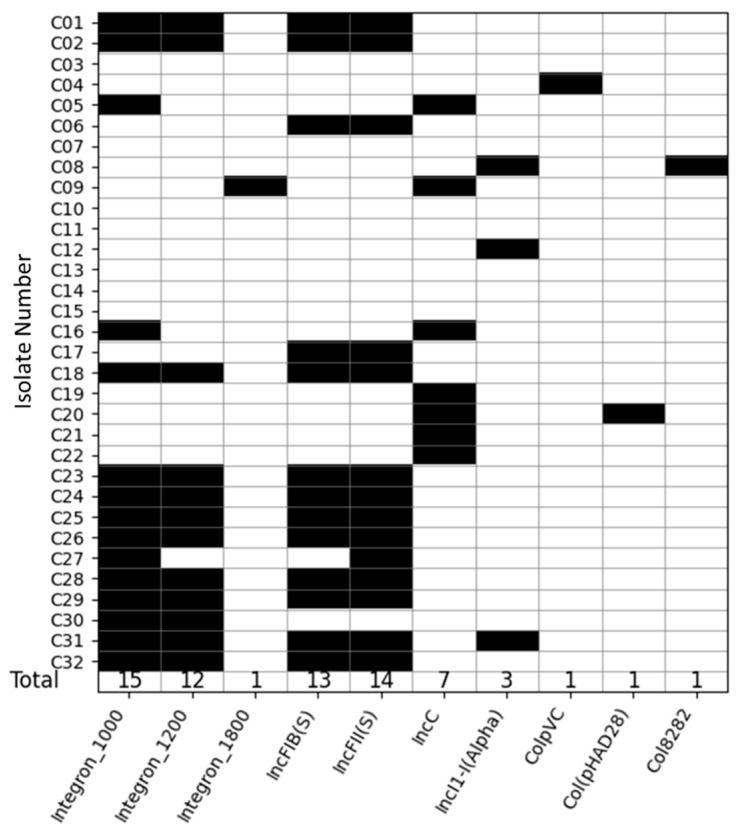
Distribution of integrons and plasmid types across *S.* Typhimurium isolates (n = 32). Each row represents the integrons and plasmids contained in one *S.* Typhimurium isolate. Black boxes indicate the presence of the integron or plasmid and white boxes indicate absence. Numbers in the final row indicate the total number of isolates with each genetic element. The first three columns refer to 1000 bp, 1200 bp, and 1800 bp integrons, respectively, and the remaining columns refer to plasmid types identified among the isolates.

Figure 1 shows the distribution of integron and plasmid types across *S.* Typhimurium isolates. Fifty percent (n = 16) of the 32 *S.* Typhimurium isolates contained integrons. Among those 16 isolates, 18.8% (n = 3) exclusively contained 1000 bp integrons, 75% (n = 12) possessed both 1000 bp and 1200 bp integrons, and only one isolate contained an 1800 bp integron.

The identification process revealed a diverse array of plasmid profiles across the isolates. Among the 32 *S.* Typhimurium isolates, 75% (n = 24) carried plasmids, with 45.5% of these (n = 15 of the total isolates) also harboring at least one integron. Seven distinct plasmid types were identified across all isolates (Figure 1). Among the 24 of 32 isolates containing plasmids, 37.5% (n = 9/24) possessed only a single plasmid, 58.33% (n = 14/24) contained two plasmids each, and one isolate had three plasmids. Specifically, 43.75% (n = 14) of all isolates harbored the IncFII (S) plasmid (Accession No. CP000858), 40.62% (n = 13) possessed the IncFIB(S) plasmid (Accession No. FN432031), and 21.87% (n = 7) contained the IncC plasmid (Accession No. JN157804). The Inc1-I(Alpha) plasmid (Accession No. AP005147) was present in 9.37% (n = 3) of the isolates. Additionally, the plasmids ColpVC, Col(pAHAD28), and Col8282, with Accession No’s JX133088, KU674895 and DQ995352, respectively, were each detected in only one isolate (Figure 1).

### 2.3. Identification of Antimicrobial Resistance Genes

A total of 22 types of AMR genes were identified across all 32 *S*. Typhimurium isolates. Aminoglycoside, fluoroquinolone, sulfonamide, and tetracycline resistance genes were each identified in 62.5% (n = 20) of isolates, while beta-lactam resistance genes were present in 65.62% (n = 21) and trimethoprim resistance genes in 9.37% (n = 3). No macrolide resistance genes were detected in the genomes or plasmids of any isolates. Among aminoglycoside resistance genes, *aadA2* was the most frequently detected, present in 43.75% (n = 14) of isolates, followed by *aph(3″)-Ib* and *aph(6)-Id*, each found in 21.87% (n = 7) of isolates. Less prevalent genes included *aac(3)-VIa*, *aadA1*, *aadA6*, and *ant(2″)-Ia*, each identified in 3.12–6.25% (n =1–2) of isolates only. For beta-lactam resistance, *bla_CARB-2_* was the most common, identified in 37.5% (n = 12) of isolates, followed by *bla_CMY-2_* in 25% (n = 8) of isolates, while *bla_TEM-1_* and *bla_CMY-132_* were present in 12.5% (n = 4) and 3.12% (n = 1) of isolate(s), respectively. Trimethoprim resistance was rare, with *dfrA12* detected in only 3.12% (n = 1) of isolates. Among fluoroquinolone resistance genes, *floR* was the most frequently identified AMR gene overall, present in 59.37% (n = 19) of isolates. Sulfonamide resistance genes *sul1* and *sul2* were found in 50% (n = 16) and 21.87% (n = 7) of isolates, respectively. Tetracycline resistance genes showed variable distribution, with *tet(G)* detected in 37.5% (n = 12) of isolates, *tet(A)* in 25% (n = 8) of isolates, and *tet(B)* and *tet(M)* each identified in only 3.12% (n = 1) of isolates. The *qacEdelta1* gene, which confers resistance to quaternary ammonium compounds, was highly prevalent, found in 50% (n = 16) of the isolates.

### 2.4. Association of Integrons and AMR Genes

**Figure 2:**
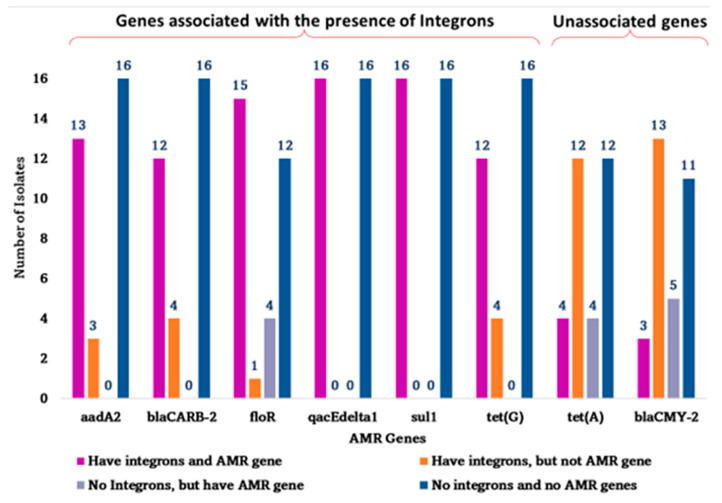
Association between presence of Integrons and AMR genes in Salmonella Typhimurium (n = 32) isolated from cattle. Genes on the left (aadA2, bla_CARB-2_, floR, qacEdelta1, sul1, tet(G)) showed significant co-occurrence with integrons (*p* < 0.05). In contrast, tet(A) and bla_CMY-2_ were not significantly associated with integrons (*p* > 0.05).

As shown in Figure 2, the statistical analysis showed significant positive associations between the presence of integrons and *aadA2*, *bla_CARB-2_*, *floR*, *qacEdelta1*, *sul1 and tet(G)* resistance genes (p < 0.05 for all genes). These genes were predominantly detected in integron-positive isolates, suggesting a possible pattern of co-occurrence. In contrast, no significant association was found between the presence of integrons and *bla_CMY-2_* and *tet(A)* genes; these genes appeared more frequently in integron-negative isolates.

### 2.5. Location of Integrons and AMR Genes

**Figure 3:**
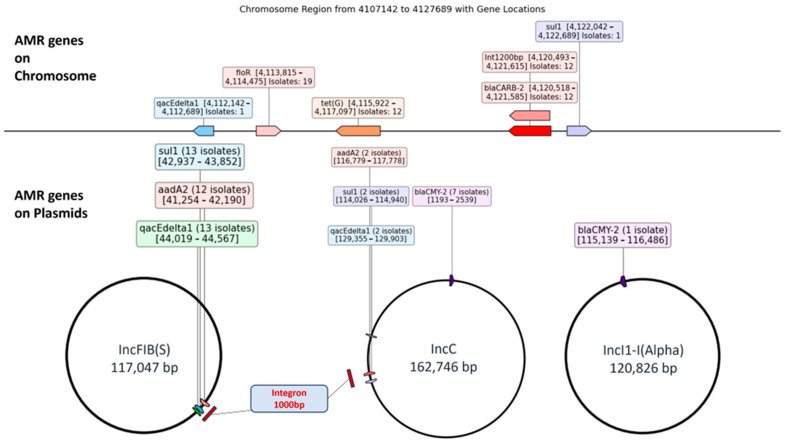
Genomic location of AMR genes in relation to integrons 1000 bp and 1200 bp. in *Salmonella* Typhimurium from cattle. Chromosomal positions are averaged due to variations in genome lengths. Integrons of 1000 bp were primarily located on the IncFIB(S) plasmid, while 1200 bp integrons were found exclusively on the chromosome. AMR genes aadA2, qacEdelta1, and sul1 were predominantly plasmid-associated near 1000 bp integrons, whereas blaCARB-2, floR, and tet(G) were chromosomally located near 1200 bp integrons. blaCMY-2 was found on plasmids distant from integrons. Different colors for AMR gene labels are used to distinguish them from each other.

Among the 15 isolates harboring a 1000 bp integron, 80% (n = 12) exhibited the integron on the IncFIB(S) plasmid, 13.3% (n = 2) on the IncC plasmid, and 6.6% (n = 1) on the chromosome. In all 12 isolates containing a 1200 bp integron, the integrons were located on chromosomes. Statistical analysis indicated a significant (*p* < 0.05) association between integron size and their location on plasmids versus chromosomes. The 1800 bp integron, present in only one isolate, was located on the IncC plasmid in that isolate. The locations of highly prevalent AMR genes were mapped with respect to integrons, showing clear patterns of distribution between chromosomes and plasmids (Figure 3).

Among 6 AMR genes associated with integrons, *aadA2* (n = 14) were primarily located within integrons 1000 bp, which were present on IncFIB(S) plasmids in 12 isolates and on IncC plasmids in 2 isolates. Similarly, *bla_CARB-2_* (n = 12) was located within 1200 bp integrons, all of which were chromosomally located in the 12 corresponding isolates. *floR* (n = 19) and *tet(G)* (n = 12) were also exclusively located on the chromosome in all isolates in which identified. The other two associated genes, *qacEdelta1* and *sul1*, were primarily associated with IncFIB(S) plasmids in 13 isolates each, with fewer occurrences on IncC plasmids (2 isolates each) and the chromosome (1 isolate each) closer to integrons 1000 bp and 1200 bp. In contrast, among non-associated genes, *bla_CMY-2_* was predominantly associated with IncC plasmids in seven isolates, with one additional occurrence on an IncI1-I(Alpha) plasmid away from either of integrons. Similarly, *tet(A)* (n = 8), exclusively localized on the chromosome, was present far away from the integrons. Therefore, it is notable that the AMR genes that have an association with integrons are present closer to the integrons on the plasmids and chromosome.

### 2.6. Phylogenetic Analysis

**Figure 4:**
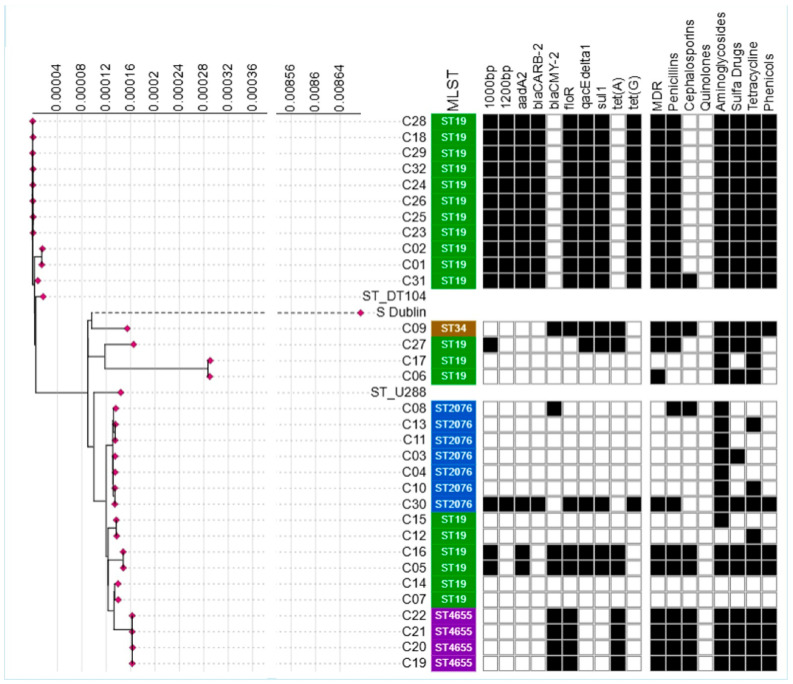
Phylogenetic tree based on the core genome of *Salmonella enterica* serovar Typhimurium isolates from cattle. Reference strains *S.* Typhimurium DT104 (ST_DT104) and U288 (ST_U288) are included for comparison, with *Salmonella* Dublin (*S*. Dublin) serving as an outgroup. Each isolate is indicated by a red diamond on the tree, with corresponding isolate numbers shown on the x-axis. Multilocus sequence type (MLST) denotes sequence types and represented by different colors only to distinguish them. Heatmaps indicate the binary presence (black) or absence (white) of integrons, antimicrobial resistance (AMR) genes, multidrug resistance (MDR), and phenotypic resistance to various antibiotic classes.

Phylogenetic analysis based on the core genome (Figure 4) showed that the majority (90.6%) of the isolates (n = 29/32) exhibited a high degree of similarity, clustering within a genetic distance of less than 0.016%. However, these genetically similar isolates were classified into different sequence types (STs), including ST19, ST34, and ST2076. In contrast, three isolates that showed greater genetic divergence still belonged to the same STs as their closest relatives. The inclusion of *Salmonella* Dublin as an outgroup confirmed its distinct phylogenetic position (0.86% difference in core genome) relative to Typhimurium isolates, while the reference strains *S.* Typhimurium DT104 and U288 (accession CP003836.1) provided context for sub-lineage differentiation within the same serovar. The DT104 reference clustered with the ST19 isolates, whereas the U288 reference grouped with a separate subset of isolates, showing internal diversity within the *S.* Typhimurium lineage. Despite the overall conservation of the core genome across different sequence types, there was significant variability in AMR gene content and phenotypic resistance even among isolates of the same ST. For example, within the ST19 sequence type, isolates exhibited different patterns of *aadA2*, *tet(A)*, and *tet(G)* presence, and this genetic variation was reflected in their tetracycline resistance phenotypes. Similarly, ST2076 isolates showed variability in the presence of *bla_CMY-2_* and cephalosporin resistance. These differences in resistance profiles within the same sequence type (ST) provided the foundation to evaluate the association between the presence of AMR genes and integrons.

## 3. Discussion

Given the increasing global concern over antimicrobial resistance, understanding the genetic factors contributing to resistance in *Salmonella* is essential for developing effective control strategies [22]. The results of our study provide a detailed genetic analysis of antimicrobial resistance (AMR) genes and integrons in *S. Typhimurium* isolates of bovine/cattle origin, retrieved from two different veterinary diagnostic laboratories in the USA. Specifically, we analyzed the distribution of integrons and AMR genes in these isolates, identifying significant associations between integron presence and specific AMR genes, as well as mapping the locations of AMR genes, in relation to integrons, on plasmids and chromosomes. After the sequencing and genomic assembly, we confirmed the serotype of 32 isolates as *S.* Typhimurium using SeqSero2 [23], with one isolate identified as *Salmonella* Agona. Among the 32 *S. Typhimurium* isolates, class I integrons were identified in 50% of the isolates, while 75% of the isolates carried plasmids. There was a widespread occurrence of AMR genes across various resistance classes, including aminoglycosides, beta-lactams, and tetracyclines. Phylogenetic analysis indicated that while isolates were genetically similar, there was notable variability in their AMR gene content and resistance phenotypes, even within the same sequence types (STs).

As class I integrons play a major role in AMR in *Salmonella* spp. compared to class II integrons [24], we therefore targeted the identification of class I integrons in this study and found them to be prevalent in 50% of the isolates. Previous studies have reported that class I integrons are prevalent in 22–55% of Gram-negative bacteria isolated from clinical settings, including *Campylobacter*, *Enterobacter*, *Escherichia, Klebsiella*, *Pseudomonas*, *Salmonella*, *Shigella*, and *Vibrio* [25]. A study by Asgharpour et al. (2014) [26] found a 36% prevalence of class I integrons in *Salmonella Infantis* isolates from chicken. However, *S.* Typhimurium has been shown to harbor more integrons than other *Salmonella enterica* strains and Antunes (2006) [27] identified integrons in 72.1% of sulfonamide-resistant *S.* Typhimurium. Another study found class I integrons in 40.4% of *S.* Typhimurium isolated from different sources including humans and the environment, either alone (4.7%) or in association with *Salmonella* genomic island 1 (SGI1) (35.7%) [28]. Our findings are consistent with previous studies, which showed a high prevalence of class I integrons in *S.* Typhimurium.

According to our study, the most prevalent AMR genes identified from respective classes in *S* Typhimurium from cattle were *floR* (59.37%) for phenicol, *sul1* (50%) for sulfonamide, *aadA2* (43.75%) for aminoglycoside, *tet(G)* (37.5%) for tetracycline, and *bla_CARB-2_* (37.5%) for beta-lactam resistances. A similar resistome has been reported in *S.* Typhimurium isolates from human stool in the USA, where IncFIB(S) and IncFII(S) plasmids were detected in all isolates, consistent with our findings of these plasmids as abundant elements [29]. Similarly, *sul1* was the most frequent mechanism of resistance to sulfonamides in *Salmonella enterica* isolated from humans, food sources and the environment in Portugal [27]. Regarding sulfonamide resistance, our findings align with the literature review, where *sul1* was the most frequently detected gene in *Salmonella enterica*, including *S.* Typhimurium from chicken and porcine sources as well; however, the predominant tetracycline resistance gene as *tet(A)* contrasts with our results [30] and could be host-associated variations in resistance gene distribution. The resistome pattern in our study aligns more closely with *S.* Typhimurium isolates from poultry than cattle in National Antimicrobial Resistance Monitoring System (NARMS) in the USA, and the prevalence of IncFIB(S) and IncFII(S) plasmids instead of IncA/C plasmids in NARMS isolates, along with the frequent detection of class 1 integrons (intI1), suggests potential shifts in plasmid- and integron-mediated AMR dissemination [31]. Hence, we need to consider continuous surveillance of resistance determinants in livestock-associated *S.* Typhimurium to better understand their evolution and public health impact.

Moreover, given the identification of integrons and a resistome pattern similar to poultry isolates from NARMS, it was important to explore the association between integrons and AMR genes, as well as their genomic locations in *S*. Typhimurium isolates from cattle, to determine their true relationship with AMR genes. In our study, AMR genes identified as the most abundant for each antibiotic class in *S*. Typhimurium isolates from cattle, were significantly associated with the presence of class I integrons. Previous studies have reported significant associations between class I integrons and the *sul1* gene in *Salmonella* isolates from various sources [32], as well as the co-existence of class I integrons and *qacEΔ1* in *S*. Typhimurium isolates from swine and poultry [33]. Similarly, *bla_CARB-2_* has also been associated with integrons in *S.* Typhimurium isolated from human stools in the USA [29].

In our study, *aadA2* was consistently found within 1000 bp integrons on plasmids, while *bla_CARB-2_* was exclusively located within 1200 bp integrons on the chromosome of *S.* Typhimurium. Similarly, the *bla_CARB-2_* was also located within a chromosomal class 1 integron in *Acinetobacter pittii* [34], suggesting a potentially conserved mechanism for the chromosomal integration of this resistance gene across different bacterial species. However, our study specifies the association of the *bla_CARB-2_* gene with 1200 bp class I integron on the chromosome. Furthermore, the *bla_CARB-2_* gene has also been identified within an integron in clinical isolates of *S. Typhimurium*, with the same study reporting the presence of the *aadA2* gene within the integron [35], which indicates the role of integrons in facilitating the horizontal transfer of aminoglycoside resistance. We found *floR* and *tet(G)* were strictly chromosomal and *qacEΔ1* and *sul1* were primarily associated with IncFIB(S) plasmids, with additional occurrence on IncC plasmids and the chromosome, but closer to 1000 bp and 1200 bp integrons in the genome. However, the *bla_CMY-2_* and *tet(A)* genes, which were not associated with integrons, were located at a distance from the integrons. This difference suggests varied mechanisms of resistance dissemination, with *bla_CMY-2_* being present as a conserved region across different plasmid types in *Salmonella* [36]. Plasmid-borne integrons (1000 bp) identified on IncFIB(S) plasmids in our study are key facilitators of horizontal gene transfer (HGT), which enables the rapid spread of AMR genes across bacterial populations, especially in livestock settings [17]. Their mobility allows resistance to disseminate not only within *S.* Typhimurium but also to other Enterobacteriaceae through conjugation [37]. In contrast, integrons located on chromosomes in our study (1200 bp) are inherited vertically during replication for stable maintenance of resistance traits over time [38]. This combination of plasmid- and chromosome-associated integrons supports their spread via plasmids and long-term persistence via chromosomal integration. These mechanisms are particularly relevant in cattle production systems, where antibiotic exposure and environmental pressures contribute to the ongoing evolution and dissemination of AMR [39].

Cattle serve as a major reservoir for *Salmonella*, making them a critical focus for studying antimicrobial resistance (AMR) in foodborne pathogens [40]. As food-producing animals, cattle have direct and indirect interactions with humans through meat consumption, dairy products, and environmental exposure, facilitating the transmission of AMR *Salmonella* strains. The spread of resistant *Salmonella* poses a significant public health risk, as infections in humans may become difficult or even impossible to treat with standard antibiotics. This highlights the importance of our findings on AMR determinants in *S.* Typhimurium, particularly given the role of mobile genetic elements in horizontal gene transfer, which can drive the dissemination of resistance genes across species and environments [41]. The findings of the study will enhance our understanding of the mechanisms driving the spread of AMR within the food animal production environment, particularly highlighting the role of cattle in the transmission of resistant *Salmonella* strains through the food chain and environmental reservoirs.

However, certain limitations should be considered while interpreting the results. First, the study was based on a relatively small sample size (n = 32), which limits the statistical power and generalizability of our findings to *Salmonella* populations in other hosts. Second, although phenotypic resistance data were included and visualized alongside genotypic data (Figure 4), we did not perform functional validation such as gene expression analysis, to confirm whether the identified AMR genes and integrons would translate into functional resistance. Moreover, another limitation of this study was the unavailability of metadata for the isolates, including the methods by which each was typed as *S.* Typhimurium. To overcome this limitation, we subjected each isolate to slide agglutination as well as evaluated the WGS for in silico serotyping alongside its sequence type and core genome phylogeny. This approach revealed high genetic similarity among 90.6% of the isolates, despite their distribution across different sequence types, with variability in AMR genes and phenotypic resistance within the same serotype (ST) (Figure 4). These findings formed the basis for reporting the association between the presence of AMR genes and integrons. However, we should focus on larger-scale studies to assess the prevalence and diversity of integrons in *Salmonella* and other bacterial strains across different regions and hosts in the future. The unavailability of metadata also limited our capacity to rule out potential sampling bias, including the health status of the animals sampled from and their history of antimicrobial use. Furthermore, we relied on a single AMR gene database for annotation, which may have excluded less common or novel resistance determinants. Finally, as this is a cross-sectional genomic study, causality cannot be inferred, our results reflect associations, not mechanistic relationships.

We need to understand how AMR spreads, particularly through plasmid-mediated transfer [17], while also developing enhanced surveillance protocols and rapid diagnostic tools for early detection [1]. At the same time, efforts to mitigate AMR should prioritize responsible antibiotic use, routine plasmid tracking, alternative treatment strategies, and targeted policy interventions, along with stronger biosecurity measures to reduce its impact in agricultural, public health and environmental settings [39].

## 4. Materials and Methods

### 4.1. Study Design and Sample Collection

This study followed the methodology outlined in a previous study by Rao et al. 2020 [18]. Briefly, different veterinary diagnostic laboratories across the United States were contacted to collect *S.* Typhimurium from their biorepositories, which included samples isolated from cattle between 2009–2012 (Supplementary File S1; Table S1). Isolates were selected through convenience sampling based on availability. The participating institutions were the Veterinary Diagnostic Laboratories at Colorado State University (CSU) and the University of Pennsylvania.

### 4.2. S. Typhimurium Identification and Antimicrobial Sensitivity Testing

A total of 33 *S.* Typhimurium isolates from cattle were streaked onto trypticase soy agar plates supplemented with 5% sheep blood (Becton, Dickinson and Company, Franklin Lakes, NJ, USA) and incubated overnight at 37 °C under ambient atmospheric conditions. The isolates were confirmed as serogroup B *Salmonella* using traditional slide agglutination (BD Diagnostic Systems^®^, Becton, Dickinson and Company) with manufacturer protocols. Following this, isolated colonies were inoculated into 1 mL of trypticase soy broth and incubated overnight at room temperature.

### 4.3. Antimicrobial Susceptibility Testing

Antimicrobial susceptibility testing (AST) was performed in the laboratory of Sangeeta Rao at the Colorado State University College of Veterinary Medicine using the Kirby–Bauer disk diffusion assay with standard procedures and zones of inhibition for susceptible, intermediate, and/or resistant phenotypes established by the Clinical Laboratory Standards Institute [42]. Antimicrobial drugs and potencies tested for AST were amoxicillin-clavulanate 20/10 μg (AMC); ampicillin 10 μg (AM); chloramphenicol 30 μg (C); cephalothin 30 μg (CF); ceftiofur 30 μg (CTO); enrofloxacin 5 μg (ERF); streptomycin 10 μg (S); sulfisoxazole 250 μg (SSS); tetracycline 30 μg (TE); trimethoprim-sulfamethoxazole 1.25/23.75 μg (SXT); cefoxitin 30 μg (FOX); ciprofloxacin 5 μg (CIP); florfenicol μg (FFC); gentamicin 10 μg (GM); kanamycin 30 μg (K); and nalidixic acid 30 μg (NA). *Escherichia coli* ATCC 25922 and *Staphylococcus aureus* ATCC 25923 were utilized as quality control organisms.

### 4.4. DNA Extraction

DNA extractions for molecular identification of integrons, integron sequencing and whole genome sequencing, were performed as described in the study by Kim et al. (2024) [19] with a Qiagen DNeasy Blood and Tissue Kit (Qiagen, Valencia, CA, USA) following standard protocol. Following extraction, the DNA concentration for each sample was quantified on a NanoDrop One Spectrophotometer (Thermo Fisher Scientific, Wilmington, DE, USA) and stored at –20 °C until further use in downstream analyses.

### 4.5. Integron Identification and Sequencing

For each *S.* Typhimurium isolate, integron identification and sequencing were performed using the methods described in Lucey et al. (2000) and Rao et al. (2008) [43,44], which facilitated concurrent sequencing of any AMR and virulence factor genes present within the integron cassette. Conventional polymerase chain reaction (PCR) was performed to identify the presence of class-I integrons. Primers based on the conserved 5′ and 3′ region of the class I integron, with the forward primer sequence 5′-GGC ATC CAA GCA AGC-3′ and the reverse primer sequence 5′-AAG CAG ACT TGA CCT GAT-3′, were used to amplify entire integron segments. Gel electrophoresis with 1% agarose gel was used to confirm PCR amplification of each integron [18]. Excised gel bands were stored at −20 °C until sequencing. DNA gel bands were extracted using QIAquick PCR Purification kit (Qiagen^®^, Hilden, Germany). Each DNA extract was mixed with ABI BigDye ^®^ Terminator v3.1 reagents and sequenced using an ABI 3130xL Genetic Analyzer (Applied Biosystems^TM^, Thermo Fisher, Foster City, CA, USA).

### 4.6. Whole Genome Sample Processing

*S.* Typhimurium isolates were processed for whole genome sequencing using previously described methods [19]. DNA extracts from all 33 samples were shipped to the Animal Disease Research and Diagnostic Lab of South Dakota State University (Brookings, SD, USA) on dry ice. DNA concentrations were normalized to 0.3 ng/µL, assigned unique barcode identifiers using a Nextera XT DNA Library Prep Kit (Illumina Inc., San Diego, CA, USA) and pooled as equi-volume aliquots. Sequencing was conducted on an Illumina MiSeq platform (Illumina Inc., San Diego, CA, USA) utilizing a 2 × 250 paired-end approach with V2 chemistry per an established protocol [45].

### 4.7. Whole Genome Denovo Assembly

Whole-genome sequencing data from all *S.* Typhimurium isolates were analyzed with Geneious Prime (Version 2024.0.5). Following sample import, Within BBDuk (Version 1.0) was employed to trim index sequences from each isolate using default parameters, ensuring the removal of adapter sequences, contaminants, and low-quality bases, thereby improving the accuracy and quality of the downstream genomic analysis [46]. Following trimming, each genome was assembled using a denovo, paired-ends approach using the Geneious Prime SPAdes plugin (version 3.13.0) [19]. The assembler method was set to “error correct + assemble” and the assembler mode was set to “careful mode”. Assembly quality metrics, including N50, L50, and total length, were assessed using QUAST (version 5.2.0) and are provided in Supplementary File S6.

### 4.8. Confirmation of S. Typhimurium and Sequence Typing

For additional serotype validation alongside slide agglutination, the assembled sequences of each isolate were subjected to SeqSero2 (v.1.3.1) Linux-based pipeline with default parameters in k-mer mode [23]. The input consisted of assembled contigs in FASTA format, and the output included predicted antigenic formulas and serovar assignments as per the White–Kauffmann–Le Minor scheme [23]. The multilocus sequence typing (MLST) analysis was performed using the MLST software (Supplementary File S1; Table S2), which applied the Achtman multi-locus sequence typing (MLST) method based on the sequences of seven housekeeping genes: *aroC*, *dnaN*, *hemD*, *hisD*, *purE*, *sucA*, and *thrA*.

### 4.9. Identification of Plasmids

PlasmidFinder (v.2.1.6) was set up on a Linux server following the instructions from the Center for Genomic Epidemiology [47,48]. The tool was run using the whole genome denovo assembly for each isolate through a command-line script with a 95% identity threshold and 80% minimum coverage. For the validation of these results, the plasmid sequences of identified plasmids were downloaded from NCBI and aligned with the assembled contigs using multiple alignment fast Fourier transform (MAFFT) (version 7.490) in the Geneious Prime interface.

### 4.10. Establishing Integron Genomic Locations

To determine the genomic location of integrons (i.e., chromosomal vs. plasmid), integron sequences were aligned to assembled whole-genome sequencing (WGS) contigs using MAFFT v7.490 with default parameters within Geneious Prime. Contigs aligned to integron sequences were then subjected to BLAST analysis against both chromosomal and plasmid assemblies for each isolate to infer their likely genomic origin. Moreover, these contigs were classified as plasmid-derived if they aligned with known plasmid sequences in NCBI, as identified using PlasmidFinder.

### 4.11. Identification of Antimicrobial Resistance Genes and Their Genomic Location

To identify AMR genes, assembled genomes and plasmids from each isolate were searched against the MegaRes 3.0 database using BLAST (basic local alignment search tool) with the following parameters: match mismatch score of 1–2, gap cost set to linear (open-extend), maximum E-value threshold of 10, and maximum target sequences set to 100. A positive gene match was defined by a pairwise identity threshold of ≥85% over ≥50% coverage of the reference library gene sequence, following the thresholds used in a previous study by Kim et al., 2024 [19]. These thresholds are used for such databases particularly when identifying genes in draft genome assemblies where full-length coverage may not always be achieved due to assembly quality, including contig size. Identified genes were refined by removing duplicates, retaining the gene with the highest pairwise percent identity; in case of further duplication, preference was given to the gene with the longest sequence and the lowest E-value (Supplementary File S2). To verify the location of the identified AMR genes, the corresponding nucleotide sequences were retrieved from the National Center for Biotechnology Information (NCBI) and aligned in Geneious Prime^®^ with the chromosomal and integron sequences of the respective isolates, as well as any plasmid-associated sequences. The alignment results were cross-referenced with plasmid sequences to confirm the genomic location, where contigs that aligned completely or partially with both the resistance gene and plasmid at a minimum pairwise identity of 85% were considered plasmid-located (Supplementary File S3).

### 4.12. Assessment of Genomic Diversity Through Core Genome Phylogeny

To determine the genomic relatedness of the isolates a phylogenetic tree was built using IQ-TREE2 [49]. Since not all isolates contained integrons and AMR genes, the phylogenetic tree was constructed using a core genome alignment, adapted from an analysis performed by Worley et al., 2018 [50]. First, genome annotation was performed using the Prokka pipeline [51] on the FASTA files of each isolate including reference genomes *Salmonella* Typhimurium DT104 (accession: NZ_CGDA01000001.1), U288 (accession: CP003836.1) and *Salmonella* Dublin (accession NZ_CP019179.1) as outgroup. This step generated annotated GFF3 files, which were subsequently analyzed using Roary for pan-genome analysis [52], identifying 2934 core genes shared among all isolates (Supplementary File S4). These core genes were then aligned to produce a core genome alignment file which served as input for phylogenetic tree construction using IQ-TREE2 [49], with automatic model selection. The resulting tree was saved in Newick format (.treefile) (Supplementary File S5) and visualized using the Interactive Tree of Life (iTOL) v6 platform [53].

### 4.13. Data Analysis and Visualization

A descriptive analysis was conducted to determine the frequency of chromosomes and plasmids harboring Class I integrons. The association between binary variables, specifically the presence and absence of AMR genes and integrons (1000 bp, 1200 bp and 1800 bp) were assessed using Fisher’s Exact Test. Similarly, the relationship between integron size and their genomic location (plasmid vs. chromosome) was evaluated using a Fisher’s Exact Test. All statistical analysis was performed using SAS v9.4 (SAS Institute Inc., Cary, NC, USA). Statistical significance for all analyses was determined as *p* < 0.05. Phylogenetic tree diagrams were generated using Python (version 3.13.0) with the Matplotlib version 3.8.4 library for data visualization (Supplementary File S1; Codes S1–S5).

## 5. Conclusions

This study shows the critical role of class I integrons regarding AMR in *S.* Typhimurium isolates from dairy cattle. The presence of integrons, specifically 1000 bp and 1200 bp, are significantly associated with key resistance genes such as *sul1*, *aadA2*, *bla_CARB_*_-2_, *qacEdelta1*, *tet(G)* and *floR*, which are located on both plasmids and chromosomes. The distribution of these integrons and resistance genes highlights the complexity of AMR transmission in foodborne pathogens, with plasmid-mediated resistance playing a significant role. These results emphasize the importance of understanding the genomic features of foodborne *Salmonella* to improve preharvest food safety risk assessments.

## Data Availability

Data are contained within the article and Supplementary Materials.

## Supplementary Materials

The following supporting information can be downloaded at: https://www.mdpi.com/article/10.3390/antibiotics14070633/s1, File S1 Table S1: Metadata table for the 33 *S.* Typhimurium isolates; Table S2: MLST Sequence Typing; File S2: AMR genes detection in MegaRes; File S3: Genomic location of AMR genes and integrons; File S4: Core genes shared among all isolates found while annotating the core genomes; File S5: Core genome alignment tree file; File S6: Genome assembly quality metrics for all isolates generated using QUAST.

## Abbreviations

The following abbreviations are used in this manuscript

AMR	Antimicrobial Resistance
ARG	Antimicrobial Resistance Genes
WGS	Whole Genome Sequencing
BLAST	Basic Local Alignment Search Tool
S. Typhimurium	*Salmonella enterica* serovar Typhimurium
