# Supplementary material for: Mapping Integron-Associated AMR Genes in Whole Genome Sequences of Salmonella Typhimurium from Dairy Cattle
Source: Antibiotics (Basel). 2025 Jun 21;14(7):633. doi: 10.3390/antibiotics14070633 (PMC12291841; doi:10.3390/antibiotics14070633)
Supplement: Supplementary file 1 [file antibiotics-14-00633-s001.zip › Supplementary File S1.pdf]

Table S1: Metadata table for the 33 S. Typhimurium isolates

Sr.	Initial Isolate number maintained	Source Bank No.	Isolate No. used in this paper	Source	Host	PCR date
1	AHD-41	1005-5	C1	U. Penn	Bovine	8.20.14
2	AHD-42	1008-44	C2	U. Penn	Bovine	8.20.14
3	AHD-43	0910-41	C3	U. Penn	Bovine	8.21.14
4	AHD-44	0910-30	-	U. Penn	Bovine	8.21.14
5	AHD-45	1010-22	C4	U. Penn	Bovine	8.21.14
6	AHD-46	0909-561	C5	U. Penn	Bovine	8.21.14
7	AHD-47	1111-33	C6	U. Penn	Bovine	8.21.14
8	AHD-48	0912-4	C7	U. Penn	Bovine	8.21.14
9	AHD-49	1010-5	C8	U. Penn	Bovine	8.21.14
10	AHD-50	1104-13	C9	U. Penn	Bovine	8.21.14
11	AHD-51	1107-14	C10	U. Penn	Bovine	8.21.14
12	AHD-52	1011-54	C11	U. Penn	Bovine	8.21.14
13	AHD-53	1104-40	C12	U. Penn	Bovine	8.21.14
14	AHD-54	0908-7	C13	U. Penn	Bovine	8.21.14
15	AHD-55	1007-66	C14	U. Penn	Bovine	8.21.14
16	AHD-56	1104-6	C15	U. Penn	Bovine	8.21.14
17	AHD-57	0910-43	C16	U. Penn	Bovine	8.21.14
18	AHD-58	1206-15	C17	U. Penn	Bovine	8.21.14
19	AHD-115	F1169112	C18	CSU-VDL	Bovine	8.25.14
20	AHD-116	F1174228-1768	C19	CSU-VDL	Bovine	8.25.14
21	AHD-117	F1174228-7675	C20	CSU-VDL	Bovine	8.25.14
22	AHD-118	F1174228-9265	C21	CSU-VDL	Bovine	8.25.14
23	AHD-119	F1264927	C22	CSU-VDL	Bovine	8.25.14
24	AHD-120	F1274970	C23	CSU-VDL	Bovine	8.25.14
25	AHD-121	F1276232	C24	CSU-VDL	Bovine	8.25.14
26	AHD-122	F1282215	C25	CSU-VDL	Bovine	8.25.14
27	AHD-123	9063126	C26	CSU-VDL	Bovine	8.25.14
28	AHD-124	F1018358	C27	CSU-VDL	Bovine	8.25.14
29	AHD-125	F1018328	C28	CSU-VDL	Bovine	8.25.14
30	AHD-126	F1016666	C29	CSU-VDL	Bovine	8.25.14

31	AHD-127	F1014979	C30	CSU-VDL	Bovine	8.26.14
32	AHD-128	F1179133	C31	CSU-VDL	Bovine	8.26.14
33	AHD-129	F1174228-9402	C32	CSU-VDL	Bovine	8.26.14

Table S2: MLST Sequence Typing

**Node**	**Method**	**MLST**	**Gene 1**	**Gene 2**	**Gene 3**	**Gene 4**	**Gene 5**	**Gene 6**	**Gene 7**
AHD-44	senterica_achtman_2	ST13	aroC(3)	dnaN(3)	hemD(7)	hisD(4)	purE(3)	sucA(3)	thrA(7)
AHD-115	senterica_achtman_2	ST19	aroC(10)	dnaN(7)	hemD(12)	hisD(9)	purE(5)	sucA(9)	thrA(2)
AHD-120	senterica_achtman_2	ST19	aroC(10)	dnaN(7)	hemD(12)	hisD(9)	purE(5)	sucA(9)	thrA(2)
AHD-121	senterica_achtman_2	ST19	aroC(10)	dnaN(7)	hemD(12)	hisD(9)	purE(5)	sucA(9)	thrA(2)
AHD-122	senterica_achtman_2	ST19	aroC(10)	dnaN(7)	hemD(12)	hisD(9)	purE(5)	sucA(9)	thrA(2)
AHD-123	senterica_achtman_2	ST19	aroC(10)	dnaN(7)	hemD(12)	hisD(9)	purE(5)	sucA(9)	thrA(2)
AHD-124	senterica_achtman_2	ST19	aroC(10)	dnaN(7)	hemD(12)	hisD(9)	purE(5)	sucA(9)	thrA(2)
AHD-125	senterica_achtman_2	ST19	aroC(10)	dnaN(7)	hemD(12)	hisD(9)	purE(5)	sucA(9)	thrA(2)
AHD-126	senterica_achtman_2	ST19	aroC(10)	dnaN(7)	hemD(12)	hisD(9)	purE(5)	sucA(9)	thrA(2)
AHD-129	senterica_achtman_2	ST19	aroC(10)	dnaN(7)	hemD(12)	hisD(9)	purE(5)	sucA(9)	thrA(2)
AHD-41	senterica_achtman_2	ST19	aroC(10)	dnaN(7)	hemD(12)	hisD(9)	purE(5)	sucA(9)	thrA(2)
AHD-42	senterica_achtman_2	ST19	aroC(10)	dnaN(7)	hemD(12)	hisD(9)	purE(5)	sucA(9)	thrA(2)
AHD-46	senterica_achtman_2	ST19	aroC(10)	dnaN(7)	hemD(12)	hisD(9)	purE(5)	sucA(9)	thrA(2)
AHD-47	senterica_achtman_2	ST19	aroC(10)	dnaN(7)	hemD(12)	hisD(9)	purE(5)	sucA(9)	thrA(2)
AHD-48	senterica_achtman_2	ST19	aroC(10)	dnaN(7)	hemD(12)	hisD(9)	purE(5)	sucA(9)	thrA(2)
AHD-53	senterica_achtman_2	ST19	aroC(10)	dnaN(7)	hemD(12)	hisD(9)	purE(5)	sucA(9)	thrA(2)
AHD-55	senterica_achtman_2	ST19	aroC(10)	dnaN(7)	hemD(12)	hisD(9)	purE(5)	sucA(9)	thrA(2)
AHD-56	senterica_achtman_2	ST19	aroC(10)	dnaN(7)	hemD(12)	hisD(9)	purE(5)	sucA(9)	thrA(2)
AHD-57	senterica_achtman_2	ST19	aroC(10)	dnaN(7)	hemD(12)	hisD(9)	purE(5)	sucA(9)	thrA(2)
AHD-58	senterica_achtman_2	ST19	aroC(10)	dnaN(7)	hemD(12)	hisD(9)	purE(5)	sucA(9)	thrA(2)
AHD-43	senterica_achtman_2	ST2076	aroC(10)	dnaN(7)	hemD(370)	hisD(9)	purE(5)	sucA(9)	thrA(2)
AHD-45	senterica_achtman_2	ST2076	aroC(10)	dnaN(7)	hemD(370)	hisD(9)	purE(5)	sucA(9)	thrA(2)
AHD-49	senterica_achtman_2	ST2076	aroC(10)	dnaN(7)	hemD(370)	hisD(9)	purE(5)	sucA(9)	thrA(2)

AHD-51	senterica_achtman_2	ST2076	aroC(10)	dnaN(7)	hemD(370)	hisD(9)	purE(5)	sucA(9)	thrA(2)
AHD-52	senterica_achtman_2	ST2076	aroC(10)	dnaN(7)	hemD(370)	hisD(9)	purE(5)	sucA(9)	thrA(2)
AHD-54	senterica_achtman_2	ST2076	aroC(10)	dnaN(7)	hemD(370)	hisD(9)	purE(5)	sucA(9)	thrA(2)
AHD-50	senterica_achtman_2	ST34	aroC(10)	dnaN(19)	hemD(12)	hisD(9)	purE(5)	sucA(9)	thrA(2)
AHD-116	senterica_achtman_2	ST4655	aroC(10)	dnaN(7)	hemD(684)	hisD(9)	purE(5)	sucA(9)	thrA(2)
AHD-117	senterica_achtman_2	ST4655	aroC(10)	dnaN(7)	hemD(684)	hisD(9)	purE(5)	sucA(9)	thrA(2)
AHD-118	senterica_achtman_2	ST4655	aroC(10)	dnaN(7)	hemD(684)	hisD(9)	purE(5)	sucA(9)	thrA(2)
AHD-119	senterica_achtman_2	ST4655	aroC(10)	dnaN(7)	hemD(684)	hisD(9)	purE(5)	sucA(9)	thrA(2)
AHD-127	senterica_achtman_2	ST2076	aroC(10)	dnaN(7)	hemD(370)	hisD(9)	purE(5)	sucA(9)	thrA(2)
AHD-128	senterica_achtman_2	ST19	aroC(10)	dnaN(7)	hemD(12)	hisD(9)	purE(5)	sucA(9)	thrA(2)

Code S1: Heatmap for Integrons and plasmids presence in all isolates:

```
import matplotlib.pyplot as plt

import numpy as np

import pandas as pd

from matplotlib.colors import ListedColormap, BoundaryNorm

data = {

  "Isolate ID": ["C01", "C02", "C03", "C04", "C05", "C06", "C07", "C08", "C09", "C10", "C11", "C12", "C13", "C14", "C15", "C16", 
"C17",

           "C18", "C19", "C20", "C21", "C22", "C23", "C24", "C25", "C26", "C27", "C28", "C29", "C30", "C31", "C32"],

  "Integron_1000": [1, 1, 0, 0, 1, 0, 0, 0, 0, 0, 0, 0, 0, 0, 0, 1, 0, 1, 0, 0, 0, 0, 1, 1, 1, 1, 1, 1, 1, 1, 1, 1],

  "Integron_1200": [1, 1, 0, 0, 0, 0, 0, 0, 0, 0, 0, 0, 0, 0, 0, 0, 1, 0, 0, 0, 0, 1, 1, 1, 1, 0, 1, 1, 1, 1, 1],

  "Integron_1800": [0, 0, 0, 0, 0, 0, 0, 0, 1, 0, 0, 0, 0, 0, 0, 0, 0, 0, 0, 0, 0, 0, 0, 0, 0, 0, 0, 0, 0, 0, 0],
```

```
    "IncFIB(S)": [1, 1, 0, 0, 0, 1, 0, 0, 0, 0, 0, 0, 0, 0, 0, 0, 1, 1, 0, 0, 0, 0, 1, 1, 1, 1, 0, 1, 1, 0, 1, 1],
    "IncFII(S)": [1, 1, 0, 0, 0, 1, 0, 0, 0, 0, 0, 0, 0, 0, 0, 0, 1, 1, 0, 0, 0, 0, 1, 1, 1, 1, 1, 1, 1, 0, 1, 1],
    "IncC": [0, 0, 0, 0, 1, 0, 0, 0, 1, 0, 0, 0, 0, 0, 0, 1, 0, 0, 1, 1, 1, 1, 0, 0, 0, 0, 0, 0, 0, 0, 0, 0],
    "IncI1-I(Alpha)": [0, 0, 0, 0, 0, 0, 0, 1, 0, 0, 0, 1, 0, 0, 0, 0, 0, 0, 0, 0, 0, 0, 0, 0, 0, 0, 0, 0, 0, 0, 1, 0],
    "ColpVC": [0, 0, 0, 1, 0, 0, 0, 0, 0, 0, 0, 0, 0, 0, 0, 0, 0, 0, 0, 0, 0, 0, 0, 0, 0, 0, 0, 0, 0, 0, 0, 0],
    "Col(pHAD28)": [0, 0, 0, 0, 0, 0, 0, 0, 0, 0, 0, 0, 0, 0, 0, 0, 0, 0, 0, 1, 0, 0, 0, 0, 0, 0, 0, 0, 0, 0, 0, 0],
    "Col8282": [0, 0, 0, 0, 0, 0, 0, 1, 0, 0, 0, 0, 0, 0, 0, 0, 0, 0, 0, 0, 0, 0, 0, 0, 0, 0, 0, 0, 0, 0, 0, 0],
}
df = pd.DataFrame(data)
df.set_index("Isolate ID", inplace=True)
column_colors = {
    "Integron_1000": "black",
    "Integron_1200": "black",
    "Integron_1800": "black",
    "IncFIB(S)": "black",
    "IncFII(S)": "black",
    "IncC": "black",
    "IncI1-I(Alpha)": "black",
    "ColpVC": "black",
```

```
    "Col(pHAD28)": "black",
    "Col8282": "black",
}
color_matrix = np.zeros((df.shape[0], df.shape[1], 4))  # RGBA matrix
for col_idx, col_name in enumerate(df.columns):
    color = plt.cm.colors.to_rgba(column_colors[col_name])
    for row_idx in range(df.shape[0]):
        if df.iloc[row_idx, col_idx] == 1:  # If the value is 1
            color_matrix[row_idx, col_idx] = color
fig, ax = plt.subplots(figsize=(8, 10))
heatmap = ax.imshow(color_matrix, aspect="auto")
for col_idx, col_name in enumerate(df.columns):
    ax.text(col_idx, -0.7, col_name, ha="left", va="bottom", fontsize=11, rotation=55, color="black")
ax.set_yticks(np.arange(df.shape[0]))
ax.set_yticklabels(df.index, fontsize=10)
ax.text(-1.5, len(df) // 2, "Isolate ID", ha="center", va="center", fontsize=12, rotation=90, color="black")  # Add "Isolate ID" label
totals = df.sum(axis=0)
ax.text(-2, len(df), "Total", ha="center", va="center", fontsize=15, rotation=0, color="black")  # Add "Total" label
for col_idx, total in enumerate(totals):
```

```
    ax.text(col_idx, len(df), str(total), ha="center", va="center", fontsize=16, color="black")

ax.set_xticks([])

ax.set_xticklabels([])

ax.set_xticks(np.arange(-0.5, df.shape[1], 1), minor=True)

ax.set_yticks(np.arange(-0.5, df.shape[0], 1), minor=True)

ax.grid(which="minor", color="white", linestyle='-', linewidth=2)

ax.tick_params(which="minor", size=0)  # Hide minor tick markers

plt.subplots_adjust(left=0.15, right=0.9, top=0.95, bottom=0.2)

plt.savefig("integrons_plasmids_heatmap.png", dpi=300, bbox_inches="tight")

plt.show()
```

Code S2: Mapping location of integrons and AMR genes on chromosomal part:

```
from dna_features_viewer import GraphicFeature, GraphicRecord

import matplotlib.pyplot as plt

chromosome_features = [

    GraphicFeature(start=4113815, end=4114475, strand=+1, color="#ffcccc",

                   label="floR\n [4113815-4114475]\nIsolates: 19"),

    GraphicFeature(start=4120518, end=4121585, strand=-1, color="#ff9999",

                   label="blaCARB-2\n [4120518-4121585]\nIsolates: 12"),
```

```
    GraphicFeature(start=4115922, end=4117097, strand=-1, color="#ff9966",
                   label="tet(G)\n [4115922-4117097]\nIsolates: 12"),
    GraphicFeature(start=4112142, end=4112689, strand=-1, color="#99ccff",
                   label="qacEdelta1\n [4112142-4112689]\nIsolates: 1"),
    GraphicFeature(start=4122042, end=4122689, strand=+1, color="#ccccff",
                   label="sul1\n [4122042-4122689]\nIsolates: 1"),
    GraphicFeature(start=4120493, end=4121615, strand=-1, color="#FF0000",
                   label="Integron_1200bp\n [4120493-4121615]\nIsolates: 12")
]
buffer_start = 5000
buffer_end = 5000
chromosome_start = min(feature.start for feature in chromosome_features) - buffer_start
chromosome_end = max(feature.end for feature in chromosome_features) + buffer_end
adjusted_features = [
    GraphicFeature(
        start=feature.start - chromosome_start,
        end=feature.end - chromosome_start,
        strand=feature.strand,
        color=feature.color,
```

```
        label=feature.label
    ) for feature in chromosome_features
]

chromosome_record = GraphicRecord(sequence_length=chromosome_end - chromosome_start,
features=adjusted_features)
fig, ax = plt.subplots(figsize=(16, 3))
chromosome_record.plot(ax=ax, figure_width=16)
ax.set_xlim(0, chromosome_end - chromosome_start)
ax.set_title(f"Chromosome Region from {chromosome_start} to {chromosome_end} with Gene Locations", fontsize=14)
ax.axis("off")
plt.tight_layout()
plt.savefig("chromosome_region_with_genes_and_isolates.png")
plt.show()
```

Code S3 Mapping Integrons and AMR genes on IncFIB(S) plasmid.

```
from dna_features_viewer import GraphicFeature, CircularGraphicRecord
import matplotlib.pyplot as plt
incFIB_S_features = [
```

```
    GraphicFeature(start=41254, end=42190, strand=+1, color="#ff9999", label="aadA2 (12 isolates) [41254-42190]"),
    GraphicFeature(start=42937, end=43852, strand=-1, color="#66b3ff", label="sul1 (13 isolates) [42937-43852]"),
    GraphicFeature(start=44019, end=44567, strand=-1, color="#99ccff", label="qacEdelta1 (13 isolates) [44019-44567]"),
]
incFIB_S_record = CircularGraphicRecord(sequence_length=117047, features=incFIB_S_features)
fig, ax = plt.subplots(figsize=(10, 10))
incFIB_S_record.plot(ax=ax, figure_width=10)
for circle in ax.patches:
    if isinstance(circle, plt.Circle):
        circle.set_color('magenta')
        circle.set_linewidth(3)
        circle.set_fill(False)
for feature in incFIB_S_features:
    ax.annotate(
        feature.label,
        xy=(feature.x_center, 0),
        xytext=(0, 5),
        ha='center', va='bottom',
        fontsize=8,
```

```
        color='black'
    )
ax.set_title("IncFIB(S) Plasmid AMR Gene Locations")
plt.tight_layout()
plt.savefig("incFIB_S_plasmid_amr_gene_locations_thicker_circle.png")
plt.show()
```

Code S4: Mapping Integrons and AMR genes on IncC plasmid

```
from dna_features_viewer import GraphicFeature, CircularGraphicRecord
import matplotlib.pyplot as plt
incFIB_S_features = [
    GraphicFeature(start=41254, end=42190, strand=+1, color="#ff9999", label="aadA2 (12 isolates) [41254-42190]"),
    GraphicFeature(start=42937, end=43852, strand=-1, color="#66b3ff", label="sul1 (13 isolates) [42937-43852]"),
    GraphicFeature(start=44019, end=44567, strand=-1, color="#99ccff", label="qacEdelta1 (13 isolates) [44019-44567]"),
]
incFIB_S_record = CircularGraphicRecord(sequence_length=117047, features=incFIB_S_features)
fig, ax = plt.subplots(figsize=(10, 10))
incFIB_S_record.plot(ax=ax, figure_width=10)
for circle in ax.patches:
```

```
    if isinstance(circle, plt.Circle):
        circle.set_color('magenta')
        circle.set_linewidth(3)
        circle.set_fill(False)
for feature in incFIB_S_features:
    ax.annotate(
        feature.label,
        xy=(feature.x_center, 0),
        xytext=(0, 5),
        ha='center', va='bottom',
        fontsize=8,
        color='black'
    )
ax.set_title("IncFIB(S) Plasmid AMR Gene Locations")
plt.tight_layout()
plt.savefig("incFIB_S_plasmid_amr_gene_locations_thicker_circle.png")
plt.show()
```

Code S5: Mapping of AMR gene(s) on IncI1-Alpha plasmid:

```
from dna_features_viewer import GraphicFeature, CircularGraphicRecord
import matplotlib.pyplot as plt
import numpy as np
incI1_I_alpha_features = [
    GraphicFeature(
        start=115139,
        end=116486,
        strand=-1,
        color="#66b3ff",
        label="blaCMY-2 (1 isolate) [115139-116486]"
    )
]
incI1_I_alpha_record = CircularGraphicRecord(sequence_length=120826, features=incI1_I_alpha_features)
fig, ax = plt.subplots(figsize=(10, 10))
incI1_I_alpha_record.plot(ax=ax, figure_width=10)
for patch in ax.patches:
    if isinstance(patch, plt.Circle):
        patch.set_color("lime")
        patch.set_linewidth(3)
```

```
        patch.set_fill(False)
num_ticks = 12
tick_positions = np.linspace(0, 120826, num_ticks, endpoint=False)
tick_angles = tick_positions / 120826 * 360
for tick, angle in zip(tick_positions, tick_angles):
    ax.annotate(
        f"{int(tick):,} bp",
        xy=(np.deg2rad(angle), 1),
        xytext=(10, 10),
        textcoords="offset points",
        ha="center",
        fontsize=8
    )
ax.set_title("IncI1-I(Alpha) Plasmid AMR Gene Locations")
plt.tight_layout()
plt.savefig("incI1_I_alpha_plasmid_amr_gene_locations_with_gene_label.png")
plt.show()
```
